# Using Network Pharmacology and Molecular Docking to Explore the Mechanism of Shan Ci Gu (*Cremastra appendiculata*) Against Non-Small Cell Lung Cancer

**DOI:** 10.3389/fchem.2021.682862

**Published:** 2021-06-09

**Authors:** Yan Wang, Yunwu Zhang, Yujia Wang, Xinyao Shu, Chaorui Lu, Shiliang Shao, Xingting Liu, Cheng Yang, Jingsong Luo, Quanyu Du

**Affiliations:** ^1^ Hospital of Chengdu University of Traditional Chinese Medicine, Chengdu, China; ^2^ Department of Biochemistry and Molecular Biology, West China School of Basic Medical Sciences and Forensic Medicine, Sichuan University, Chengdu, China; ^3^ Chengdu University of Traditional Chinese Medicine, Chengdu, China; ^4^ Faculty of Geosciences and Environmental Engineering, Southwest Jiaotong University, Chengdu, China

**Keywords:** Shan Ci Gu, non-small cell lung cancer, network pharmacology, molecular docking, molecular mechanism

## Abstract

**Background:** In recent years, the incidence and mortality rates of non-small cell lung cancer (NSCLC) have increased significantly. Shan Ci Gu is commonly used as an anticancer drug in traditional Chinese medicine; however, its specific mechanism against NSCLC has not yet been elucidated. Here, the mechanism was clarified through network pharmacology and molecular docking.

**Methods:** The Traditional Chinese Medicine Systems Pharmacology database was searched for the active ingredients of Shan Ci Gu, and the relevant targets in the Swiss Target Prediction database were obtained according to the structure of the active ingredients. GeneCards were searched for NSCLC-related disease targets. We obtained the cross-target using VENNY to obtain the core targets. The core targets were imported into the Search Tool for the Retrieval of Interacting Genes/Proteins database, and Cytoscape software was used to operate a mesh chart. R software was used to analyze the Gene Ontology biological processes (BPs) and Kyoto Encyclopedia of Genes and Genomes (KEGG) pathway enrichment. The core targets and active compounds were molecularly docked through Auto-Dock Vina software to predict the detailed molecular mechanism of Shan Ci Gu for NSCLC treatment. We did a simple survival analysis with hub gene to assess the prognosis of NSCLC patients.

**Results:** Three compounds were screened to obtain 143 target genes and 1,226 targets related to NSCLC, of which 56 genes were related to NSCLC treatment. Shan Ci Gu treatment for NSCLC involved many BPs and acted on main targets including epidermal growth factor receptor (EGFR), ESR1, and SRC through signaling pathways including the endocrine resistance, EGFR tyrosine kinase inhibitor resistance, and ErbB signaling pathways. Shan Ci Gu might be beneficial for treating NSCLC by inhibiting cell proliferation and migration. Molecular docking revealed that the active compounds β-sitosterol, stigmasterol, and 2-methoxy-9,10-dihydrophenanthrene-4,5-diol had good affinity with the core target genes (EGFR, SRC, and ESR1). Core targets included EGFR, SRC, ESR1, ERBB2, MTOR, MCL1, matrix metalloproteinase 2 (MMP2), MMP9, KDR, and JAK2. Key KEGG pathways included endocrine resistance, EGFR tyrosine kinase inhibitor resistance, ErbB signaling, PI3K-Akt signaling, and Rap1 signaling pathways. These core targets and pathways have an inhibitory effect on the proliferation of NSCLC cells.

**Conclusion:** Shan Ci Gu can treat NSCLC through a multi-target, multi-pathway molecular mechanism and effectively improve NSCLC prognosis. This study could serve as a reference for further mechanistic research on wider application of Shan Ci Gu for NSCLC treatment.

## Introduction

Lung cancer is a malignant tumor with the highest incidence and mortality rates in China (Zhu et al., 2020), with cough, hemoptysis, chest pain, fever, and shortness of breath being the main clinical manifestations. Over the past 50 years, the incidence and mortality rates of lung cancer have increased significantly in many countries, and it now ranks first among the causes of death from malignant tumors in China’s urban citizens. Non-small cell lung cancer (NSCLC) accounts for more than 80% of lung cancers, and the vast majority of patients are diagnosed at advanced inoperable stages. NSCLC remains the single most common malignancy of lung cancer, which has caused an increasing number of deaths in recent years (Ye et al., 2021). The main treatment methods for lung cancer include surgery, radiotherapy, chemotherapy, targeted therapy, and immunotherapy, among which concurrent radiotherapy and chemotherapy are the standard modes of treatment for NSCLC, but its five-year survival rate is only approximately 5%, and the side effects of radiotherapy and chemotherapy seriously reduce the quality of life (Cheng and Chen, 2020). In the last decade, NSCLC treatment has achieved certain efficacy through the use of targeted therapy, but all of the commonly used drugs have a single pathway and are subject to increasing drug resistance. Thus, the use of Chinese medicine in combination with the commonly used drugs and treatments can enhance the effectiveness of conventional treatment, reduce drug resistance, reduce adverse effects and toxicity, alleviate patient suffering, and improve the quality of life (Su et al., 2020).

Shan Ci Gu is the dried pseudostem of plants of the Orchidaceae family, and is sweet, slightly pungent, and cool in nature. In traditional Chinese Medicine theory, Shan Ci Gu belongs to the liver and spleen meridians. It is used to treat carbuncles and furuncles and to heal sores and phlegm ulcers, snake and insect bites, and traumatic wounds Guo and Wang, 2012. It has antibacterial, antihypertensive, gout, antitumor, and acetylcholine receptor M3-blocking effects, providing it high medicinal value (Zhang et al., 2019). Shan Ci Gu is commonly used as a clinical anti-tumor herbal medicine to treat a variety of cancers. The extracts of this herb can be used to treat Lewis lung cancer, liver cancer, and breast cancer (as well as human breast cancer MDA-MB-231 cells) (Si et al., 2020).

**FIGURE 1:**
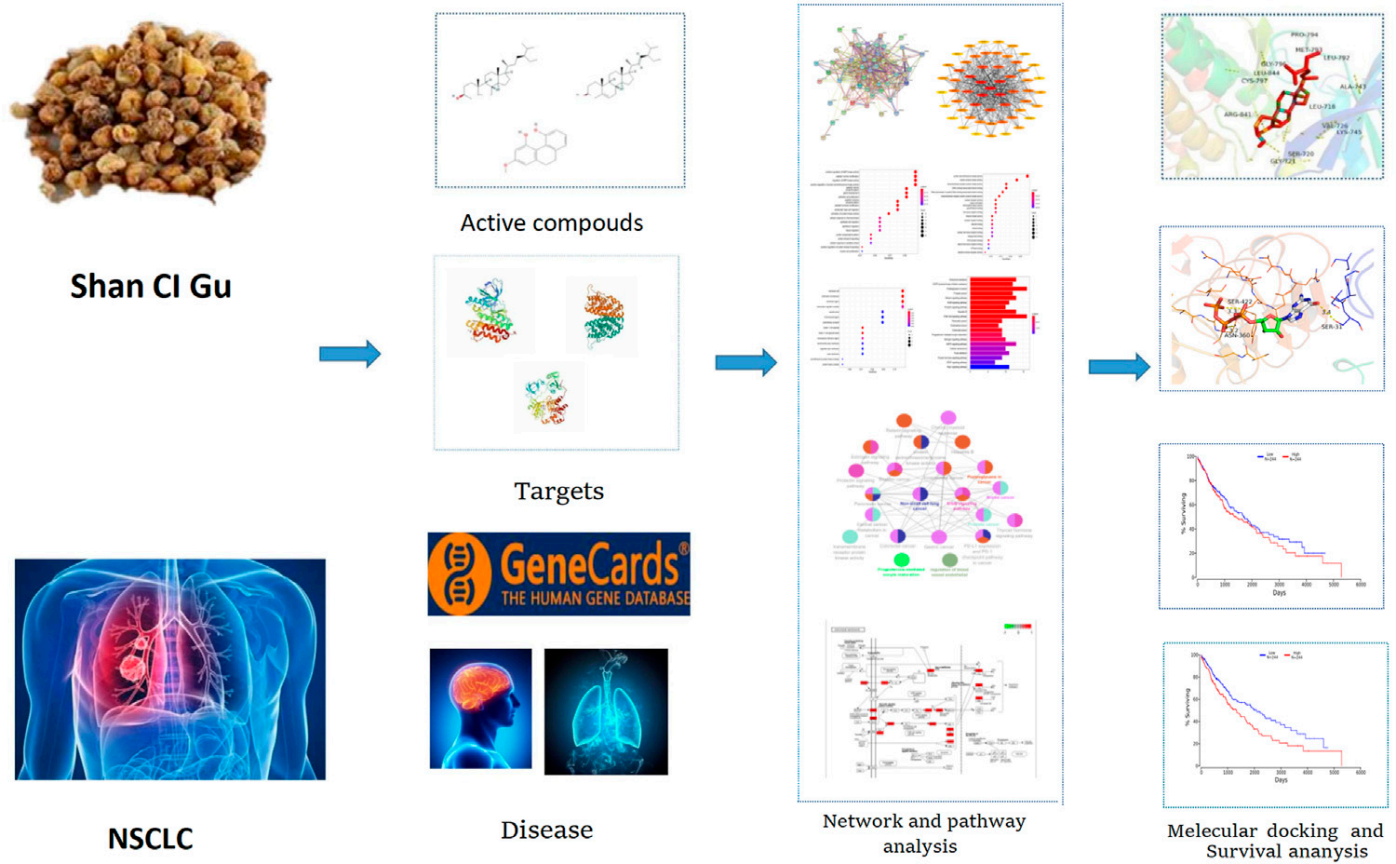
Technological roadmap.

Shan Ci Gu also has a level of clinical efficacy against NSCLC and is effective in improving the quality of life of patients with advanced NSCLC, reducing the side effects of radiotherapy and chemotherapy, as well as enhancing the sensitivity to radiotherapy and chemotherapy (Shan et al., 2015). Its extracts can inhibit the growth of tumor cells directly or indirectly through cytotoxic effects and improve the body’s immunity (Li et al., 2018). Network pharmacology is a new discipline based on the theory of systems biology, the network analysis of biological systems, and the selection of specific signal nodes (Nodes) for the design of multi-target drug molecules, which can predict the molecular mechanism of drug action in disease. Molecular docking is mainly used to study intermolecular interactions and predict the binding mode and relationship. Molecular docking is also used for drug and protein function prediction. By using network pharmacology and molecular docking, we aimed to explore the mechanism of action of Shan Ci Gu for the treatment of NSCLC to improve the condition of patients with NSCLC and reduce mortality rates. Figure 1 shows our technology roadmap.

## Methods

### Search and Collection of Active Ingredients of Shan Ci Gu

“Shan Ci Gu” was searched for in the Traditional Chinese Medicine Systems Pharmacology (TCMSP) database (http://tcmspw.com/tcmsp.php) to find multiple active compounds. Oral bioavailability (OB) represents the rate and degree of absorption of traditional Chinese medicine in the human circulatory system, while drug-likeness (DL) is the similarity of compounds to known drugs. OB ≥ 30% and DL ≥ 0.18 were used to screen the active ingredients of Shan Ci Gu. The names of the compounds collected from the TCMSP database were entered into the PubChem database (https://pubchem.ncbi.nlm.nih.gov/) and the SMILE and 3D “Standard Delay Format” (SDF) structures of the corresponding compounds were downloaded for the prediction of target genes and molecular docking.

Acquisition and collection of targets for the active ingredients of Shan Ci Gu for the treatment of NSCLC.

The SMILE structure of the active compound was uploaded into the Swiss Target Prediction database (http://www.swisstargetprediction.ch/), the predicted target gene data were downloaded in CSV format, and all active compounds were filtered and integrated using Microsoft Excel software. Predicted targets of the components were imported into UniProt for normalization and then restricted to human species, and all retrieved target proteins were corrected to their official names. The anti-cancer targets of the main components of Shan Ci Gu were imported into Cytoscape (3.7.2) to generate a “component-target” network. The nodes in the network diagram are the chemical components and targets. The correlation between components and targets is represented by edges.

Through the GeneCards database (https://www.gene cards. org/), targets related to NSCLC were searched by entering the keyword “Non-Small Cell Lung Cancer.” Then, we combined the components and targets of Shan Ci Gu with those of the drug. Predicted target mapping of the relevant targets with NSCLC targets was used to obtain the target of action of Shan Ci Gu for NSCLC. VENNY (https://bioinfogp.cnb.csic.es/tools/venny_old/) was used to draw a Venn diagram of matsutake and NSCLC targets.

### Protein-Protein Interaction Analysis and Core Target Screening

The intersection of Shan Ci Gu and NSCLC targets was uploaded to the online site of STRING version 10.5 (https://string-db.org/). The protein type was set to “*Homo sapiens*,” a high confidence level of 0.7 was selected, and the other parameters were set to default values. The protein interaction relationships were retrieved. Node1, Node2, and the combined score from the export file were imported into Cytoscape, and the interaction network was constructed. The node size reflected the degree value and the thickness of the edge reflected the combined core in the final PPI network diagram, and the core proteins with the top 10° values were selected.

### Gene Ontology and Kyoto Encyclopedia of Genes and Genomes Pathway Enrichment Analyses

GO analysis of the relevant obtained intersection target proteins was performed using R software to select the biological process (BP), cellular component (CC), and molecular function (MF). These data were plotted as bubble charts, and R software was used to construct a bar graph of the KEGG biological pathway results, collect the targets from the pathway, and upload the active components, pathways, and targets to Cytoscape 3.7.2 software to create a “component-target-pathway” map. A network diagram was constructed to visualize, and thus, explore the mechanisms of Shan Ci Gu related to the treatment of NSCLC in detail. Cluster ONE was then used to sift the candidate genes and retrieve them for further analysis. The KEGG function of the genes was further understood by using the Cytoscape plugin Clue GO, and the relevant KEGG pathways were selected for enrichment analysis. The intersecting genes were imported into R software, and the script was run to obtain a predictive map of the endocrine pathway mechanism.

### Molecular Docking

Molecular docking is a method for drug design by exploring the interaction and recognition between receptors and ligands. It is a theoretical simulation method that focuses on the study of intermolecular interactions and the prediction of their binding patterns and affinities. In recent years, molecular docking methods have become an important technique in the field of computer-aided drug research (Chen et al., 2020). AutoDock Vina is an open-source molecular docking program designed by the Scripps Research Institute for the computation of semi-flexible molecular docking. AutoDock Vina uses a complex gradient algorithm and multi-threaded techniques to make more accurate and faster predictions than AutoDock4. Semi-flexible docking means that the conformation of the ligand molecule can be changed according to the receptor molecule and is flexible, while the receptor molecule does not change and is rigid. The SDF structure of the active ingredient was imported into Chem3D 18.0 for optimization. The main protein targets selected were passed through the PDB database (https://www.rcsb.org/). Therefore, we searched the PDB database for the 3D structures of the ten potential targets of Shan Ci Gu in the treatment of NSCLC and found 3D structures for ten of the targets (EGFR, SRC, ESR1, ERBB2, MTOR, MCL1, MMP2, MMP9, KDR, and JAK2.). Then, the best protein crystal structure was selected [images with lower resolution (A) with observable ligands and a relatively intact structure were more desirable] and downloaded from the PDB database. The PDB files of the active compound and ligand molecules were imported into AutoDock Tools. We removed these target proteins’ water molecules, added polar hydrogen, and built active pockets active pockets, which were saved as PDBQT format files for later use. By adjusting target protein X-Y-Z coordinates and grid size, optimizing the position of protein structure-binding sites for molecular docking. AutoDock Vina was run to dock the treated active compound to the target protein ten times, and the lowest binding energy for each docking was taken as the final result. The complexes were then observed and plotted using PyMOL.

### Survival Analysis

We used OncoLnc (http://www.oncolnc.org/) to obtain OS (Overall Survival) and DFS (Disease-free survival) significance data for ten core genes (EGFR, SRC, ESR1 EGFR, SRC, ESR1, ERBB2, MTOR, MCL1,MMP2, MMP9, KDR, and JAK2) in all squamous lung cancers at TCGA. High (50%) and low (50%) cutoff values were used as expression thresholds to split high and low expression cohorts, and 488 samples were analyzed using log-rank test and Kaplan-Meier to obtain survival maps. *p* < 0.05 was considered a statistically significant difference.

## Results

### Search and Collection of the Active Ingredients of Shan Ci Gu

Based on the screening conditions of the compounds, three active ingredients were collected from the TCMSP database, namely, 2-methoxy-9,10-dihydrophenanthrene-4,5-diol, stigmasterol, and β-sitosterol. The details are shown in Table 1.

**TABLE 1:** Active ingredients of Shan Ci Gu.

MOL	English name	Pubchem CID	OB	DL
MOL000358	beta-sitosterol	222,284	36.91	0.75
MLL000449	Stigmasterol	5280794	43.83	0.76
MOL007991	2-methoxy-9,10-dihydrophenanthrene-4,5-diol	11506999	44.97	0.18

### Acquisition of Active Targets for the Treatment of Non-Small Cell Lung Cancer From Shan Ci Gu

**FIGURE 2:**
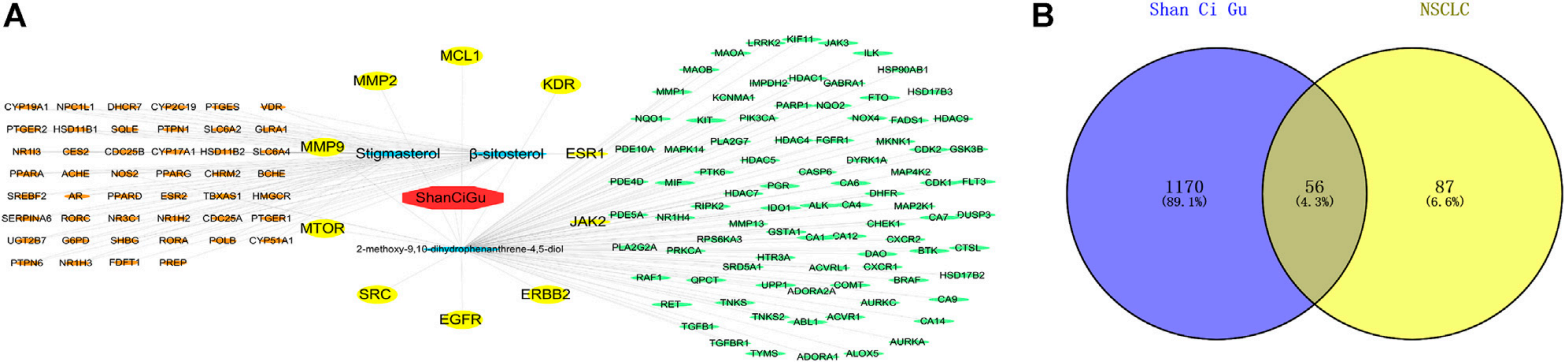
Drug-active ingredient-target network diagram and Venn diagram. Drug-active ingredient-target network diagram **(A)**. The red octagons represent the drug, the blue ovals represent the active ingredients of the drug. The yellow ovals represent the hub genes, the orange ovals represent the relevant targets of stigmasterol and β-sitosterol, and the green ovals represent the relevant targets of 2-methoxy-9,10-dihydrophenanthrene-4,5-diol. Lines represent the relationships between nodes; the more connections the nodes have, the more important they are. Venn diagram **(B)**. The blue part represents the number of drug targets, and the yellow part represents the number of disease targets.

The Swiss Target Prediction database of predicted targets was compiled, resulting in 42 predicted targets for the active ingredient β-sitosterol and 45 predicted targets for the active ingredient stigmasterol, with an active ingredient of 2-methoxy-9,10-dihydrophenanthrene-4,5-diol. The number of predicted targets for dihydrophenanthrene-4,5-diol was 100, and the total number of targets was 143 by combining the duplicates. The “drug-active compound-target” network graph built in Cytoscape 3.7.2 reflects the correspondence of the compound targets, as shown in Figure 2A. The GeneCards database was searched with the keyword “Non-Small Cell Cancer,” and 1226 NSCLC targets were obtained based on the relevance score. The 56 intersecting genes of Shan Ci Gu and NSCLC were obtained, as shown in Figure 2B.

### Protein-Protein Interaction Network Analysis

**FIGURE 3:**
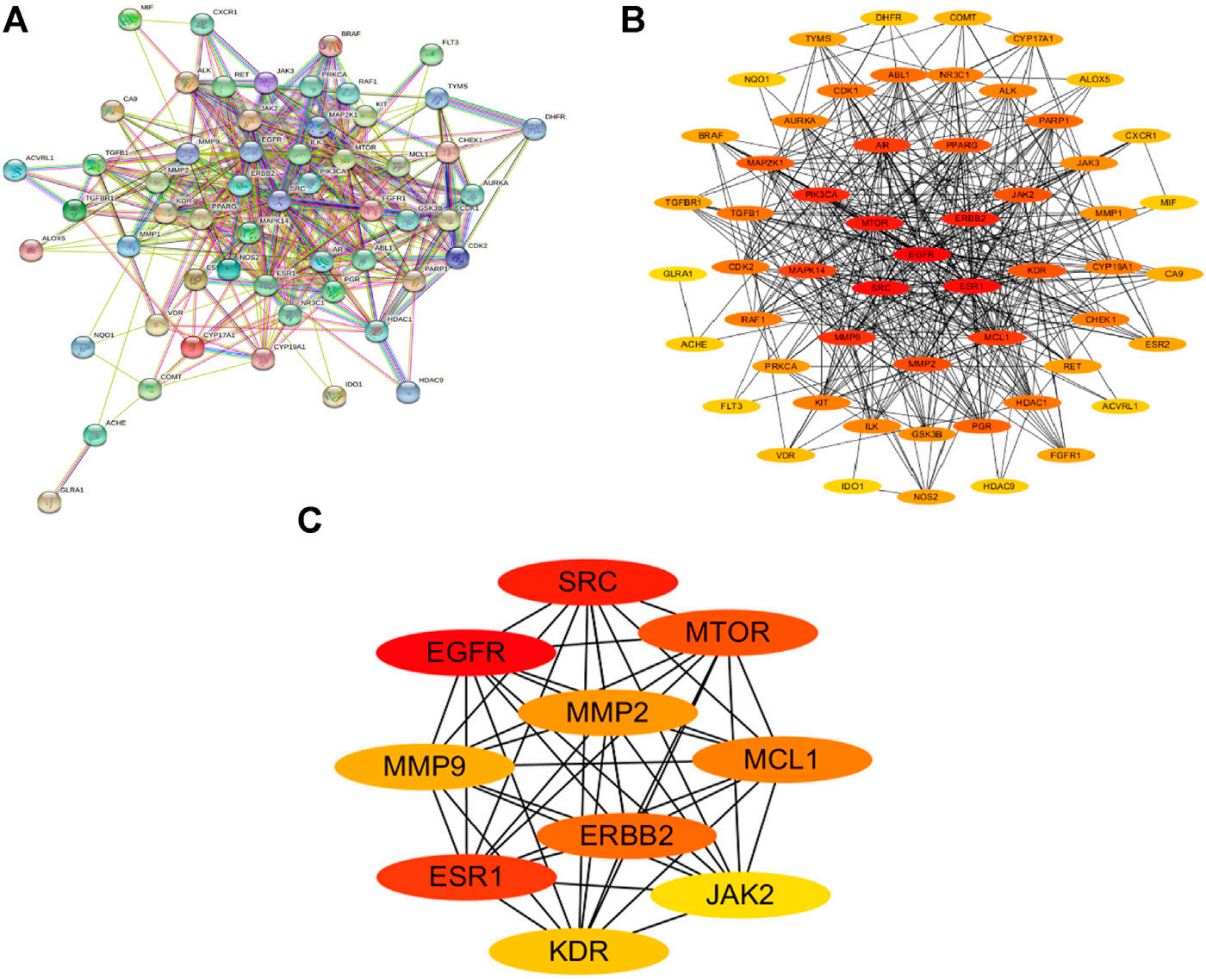
Protein-protein interaction (PPI) analysis. **(A)** The PPI network was constructed using the plug-in targets from the Search Tool for the Retrieval of Interacting Genes/Proteins database, which were imported into Cytoscape, and the targets were the candidates used for non-small cell lung cancer treatment. **(B)** Proteins are represented by nodes (colors from red to yellow illustrate the extent to which the medical targets have combined with each other). Edges indicate protein-protein associations. **(C)** The top 10 targets (hub targets) in the PPI network ranked by maximal clique centrality using the cytoHubba plug-in.

The 56 intersection targets of the predicted Shan Ci Gu and NSCLC were imported into the STRING database to select a *H. sapiens*-generated PPI network map and obtain protein interaction relationships (Figure 3A). The intersection targets were imported into Cytoscape 3.7.2 to create a network diagram of potential target interactions (Figures 3B,C). Nodes represent proteins and edges represent relationships between proteins, resulting in a total of 56 nodes and 409 edges. The colors from yellow to red represent small to large degree values, respectively, and according to the degree values, the key nodes, which are epidermal growth factor receptor (EGFR), ESR1, SRC, ERBB2, MTOR, MCL1, matrix metalloproteinase 2 (MMP2), MMP9, KDR, and JAK2, were selected for interactions with NSCLC.

### Gene Ontology Analysis and Kyoto Encyclopedia of Genes and Genomes Pathway Enrichment Analysis

**FIGURE 4:**
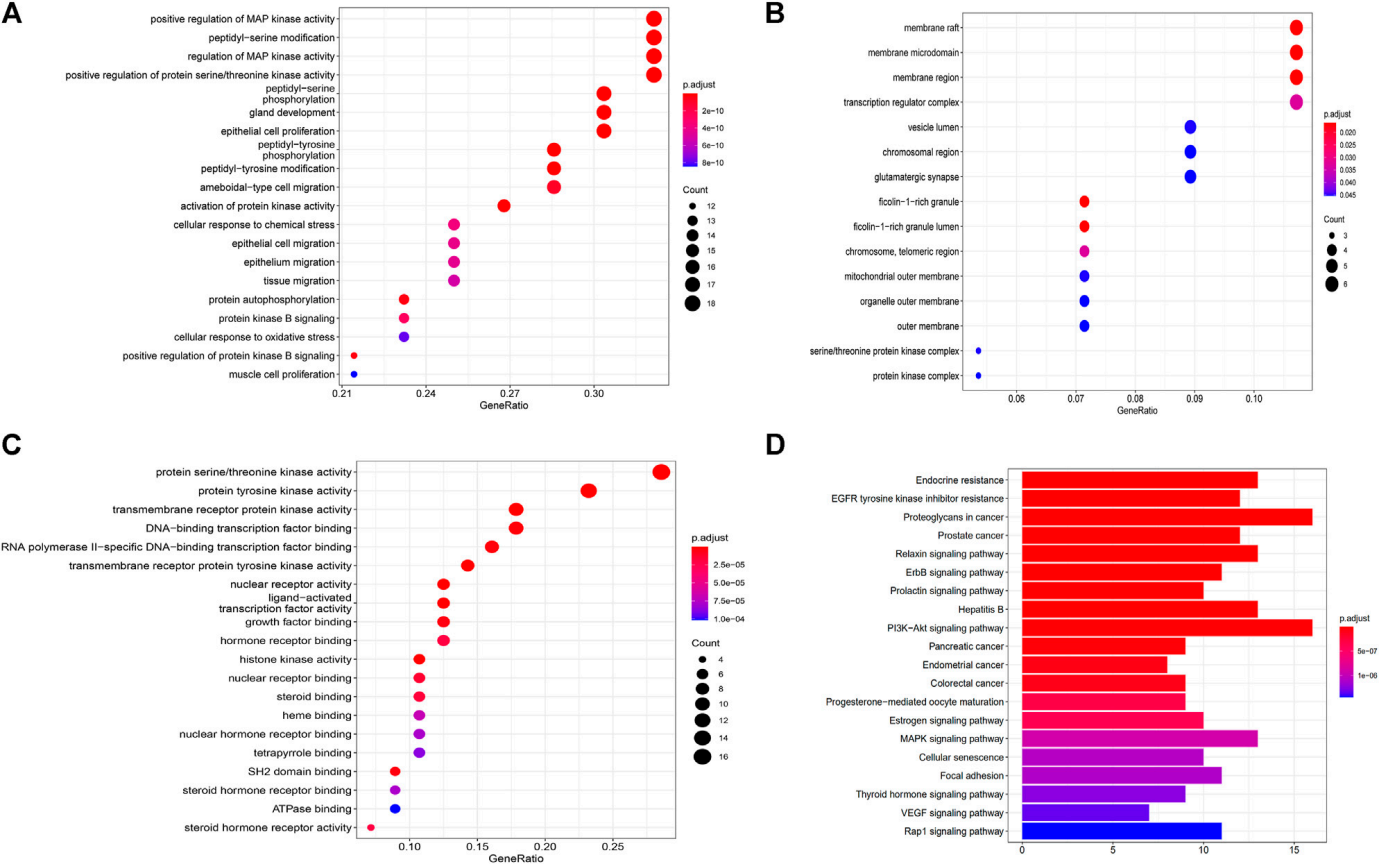
Gene Ontology and Kyoto Encyclopedia of Genes and Genomes pathway enrichment analyses. **(A–C)** The names of the biological processes, cellular component and molecular function terms distributed in the ordinate and the degree of enrichment in the abscissa. The size of the dots represents the number of genes; the larger is the dot, the higher is the number of genes in the corresponding process. **(D)** The names of the pathways distributed in the ordinate and the number of genes enriched in the pathway distributed in the abscissa. *p* values indicate the importance of enrichment; the lower is the *p* value, the redder is the color of the graph, and the higher is the enrichment.

**FIGURE 5:**
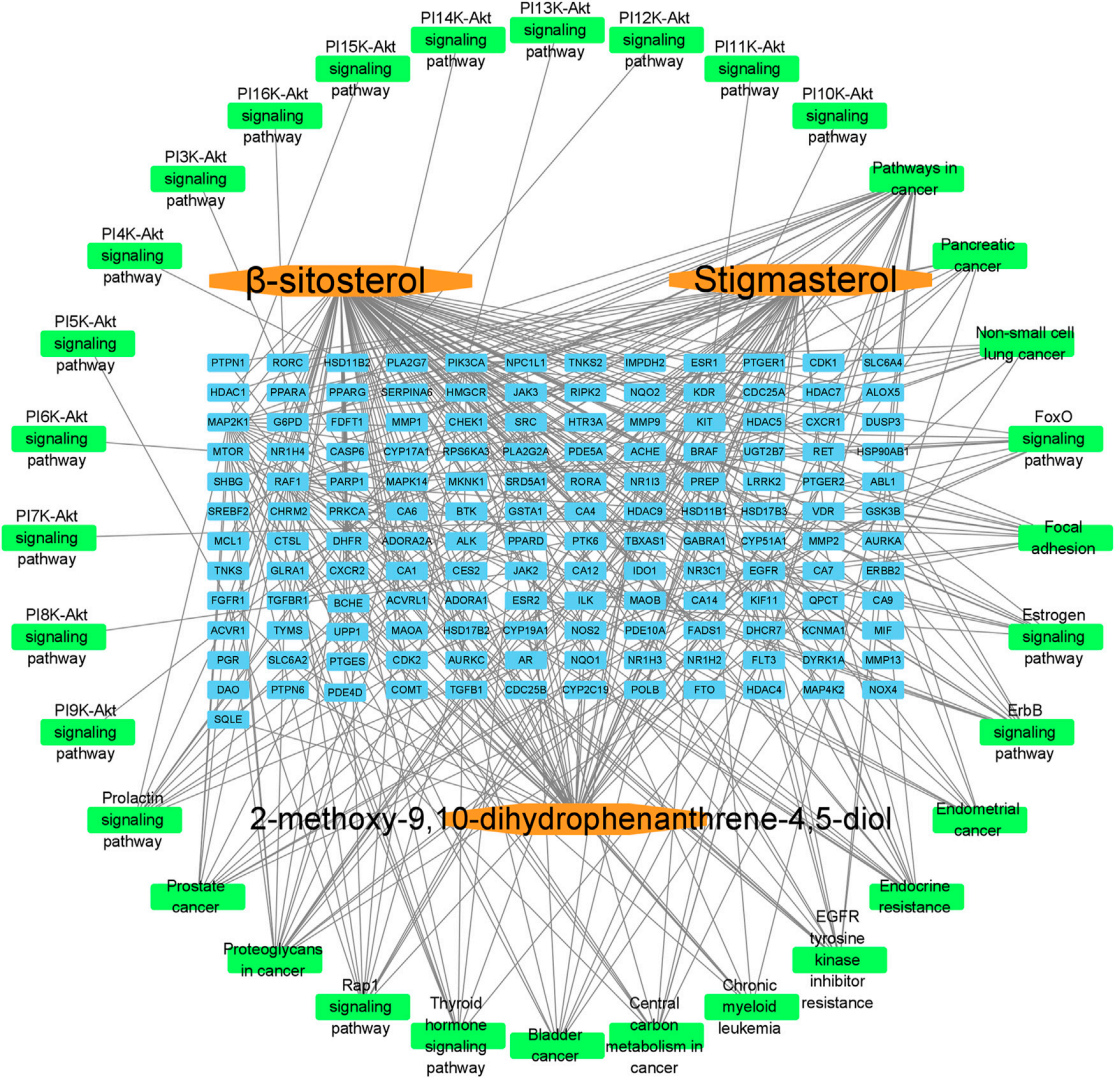
Component-target-signal pathway. The orange boxes indicate the active ingredients. The blue boxes indicate the gene names. The green boxes indicate the signal pathways.

**FIGURE 6:**
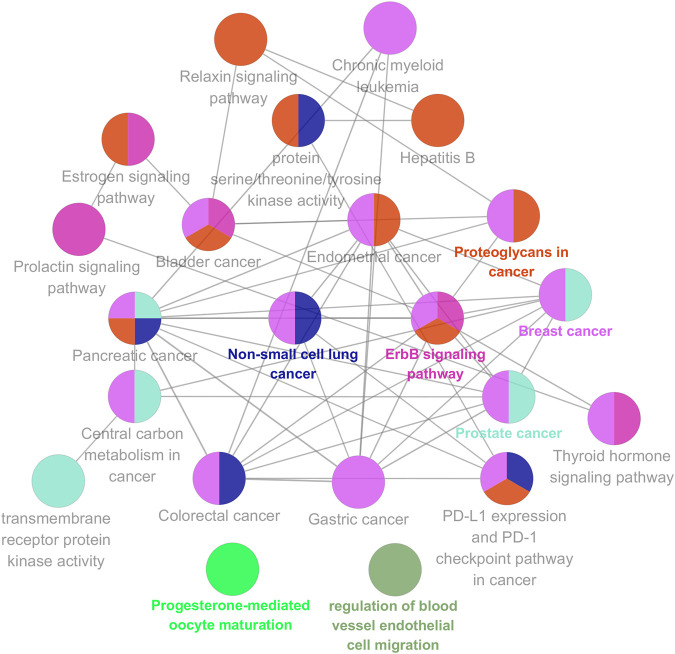
KEGG analysis using the ClueGO plug-in. Kyoto Encyclopedia of Genes and Genomes pathway analyses of the potential targets of Shan Ci Gu against non-small cell lung cancer by the ClueGO plug-in. Each node is a representative enrichment pathway. The nodes indicate the number of genes shared between pathways. The color represents the enrichment classification of the node.

**FIGURE 7:**
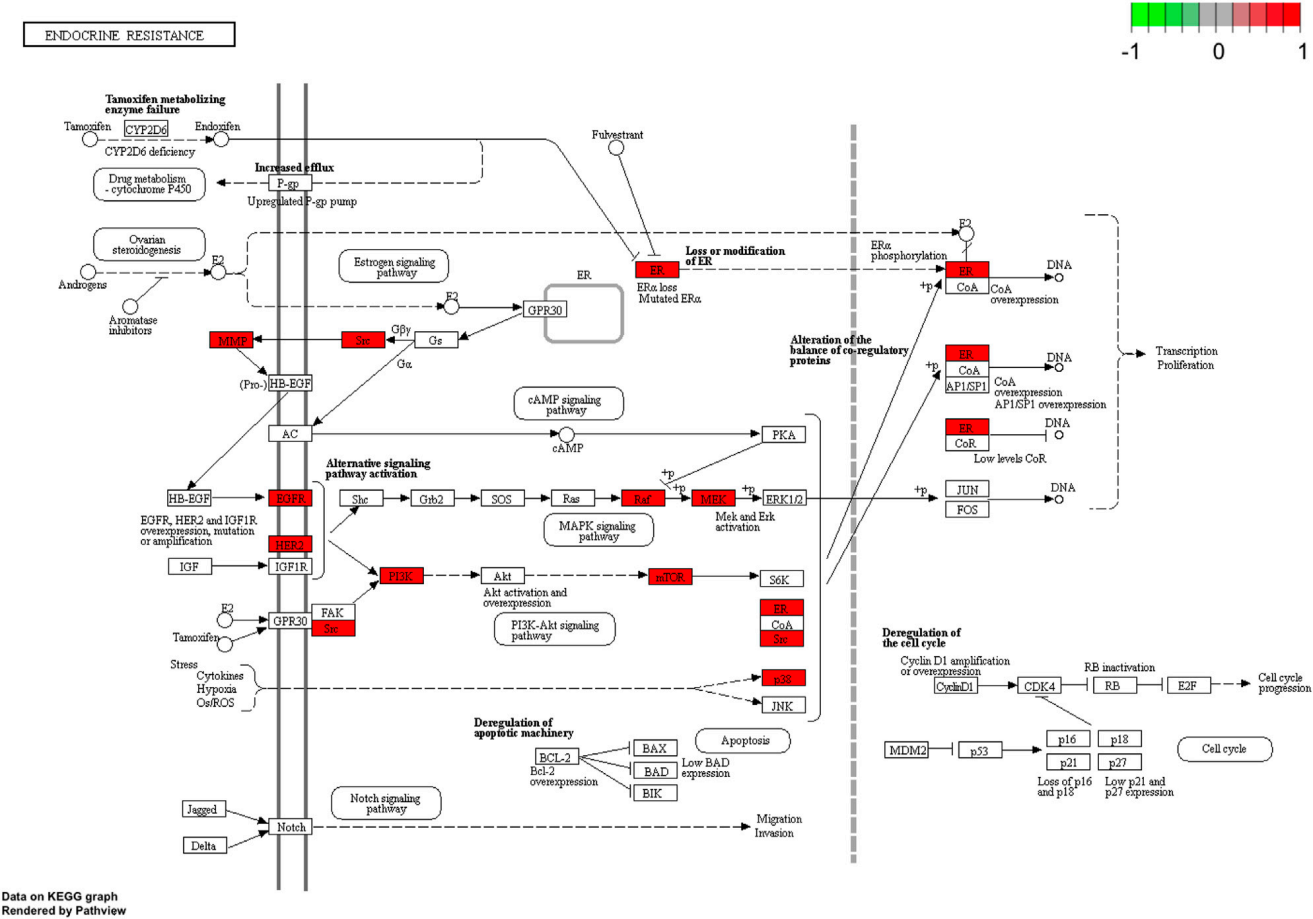
Relevant targets in the signaling pathway of Shan Ci Gu and endocrine resistance. Green and red rectangles indicate unidentified and identified proteins, respectively.

To further explore possible mechanisms of the 56 candidate targets for the treatment of NSCLC, R software was used for GO enrichment analysis with the candidate target and KEGG pathway analysis of these targets for the treatment of NSCLC with Shan Ci Gu. The results showed that the number of BP terms was 1,385, CC was 35, and MF was 69. The top 15 BPs are shown in bubble charts (Figures 4A–C). KEGG pathway enrichment analysis was conducted using R software and involved 125 terms. The bar chart (Figure 4D) reflects the top 20 entries. Detailed data are shown in Table 2. The active ingredients, candidate targets, and 20 pathways were imported into Cytoscape to create a “component-target-pathway” network diagram and visualize it (Figure 5). The predictive map of the mechanism of the endocrine pathway is shown in Figure 6. Relevant targets in the signaling pathway of Shan Ci Gu and endocrine resistance is shown in Figure 7.

**TABLE 2:** Kyoto Encyclopedia of Genes and Genomes pathway analysis for the treatment of non-small cell lung cancer, using R software.

Pathway ID	Paythway	*p*value	Number of target hits
hsa01522	Endocrine resistance	6.61E-14	13
hsa01521	EGFR tyrosine kinase inhibitor resistance	1.26E-13	12
hsa05205	Proteoglycans in cancer	2.76E-13	16
hsa05215	Prostate cancer	1.60E-12	12
hsa04926	Relaxin signaling pathway	2.48E-12	13
hsa05219	Bladder cancer	5.53E-12	9
hsa04012	ErbB signaling pathway	9.02E-12	11
hsa05224	Breast cancer	1.34E-11	13
hsa04917	Prolactin signaling pathway	3.15E-11	10
hsa05230	Central carbon metabolism in cancer	3.15E-11	10
hsa05223	Non-small cell lung cancer	4.22E-11	10
hsa05161	Hepatitis B	4.63E-11	13
hsa04151	PI3K-Akt signaling pathway	1.09E-09	16
hsa05212	Pancreatic cancer	1.87E-09	9
hsa05226	Gastric cancer	4.19E-09	11
hsa05213	Endometrial cancer	4.62E-09	8
hsa05210	Colorectal cancer	5.72E-09	9
hsa05225	Hepatocellular carcinoma	1.49E-08	11
hsa04914	Progesterone-mediated oocyte maturation	2.20E-08	9
hsa04915	Estrogen signaling pathway	2.75E-08	10

### Molecular Docking

Table 3 shows the results acquired from the molecular docking software (AutoDock Vina). Processed by PyMOL software, the docked complex and ‘Best-Docked Complex’ show images of the best docking of the receptor and ligand. According to Table 3, we can conclude that the binding energies of the three active ingredients to the top three ranked target gene (EGFR, SRC, and ESR1) transcriptional proteins were all less than—5 kcal/mol, and most of the binding energies of the remaining core target proteins were also less than 0, indicating a high affinity between the compounds and the core target genes. The binding energies of MMP2 and MMP9 to the three components were all greater than 0, indicating that the two target proteins had low binding ability to the active components of Shanzi mushroom and cannot be used as active sites.

**TABLE 3:** Molecular docking results.

Target	Target structure	Compound	Compound 2D structure	Affinity (kcal/ mol)	Best-docked complex
EGFR	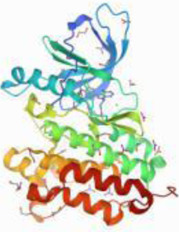	Beta-sitosterol	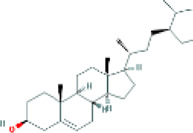	−9.0	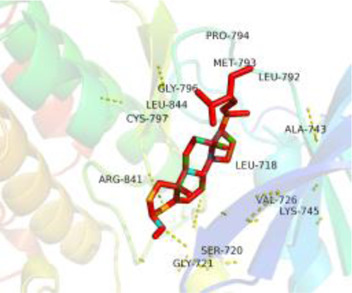
		Stigmasterol	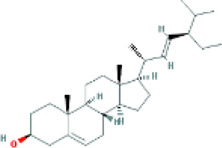	−9.1	
		2-Methoxy-9,10-dihydrophenanthrene-4,5-diol	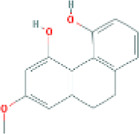	−8.0	
SRC	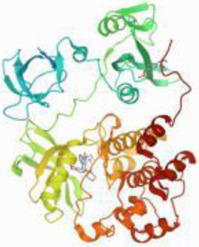	Beta-sitosterol	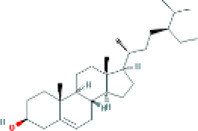	−10.1	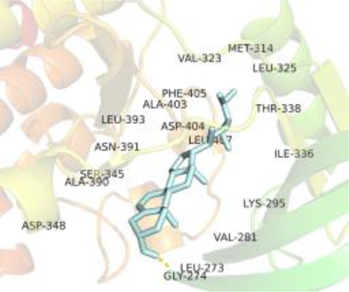
		Stigmasterol	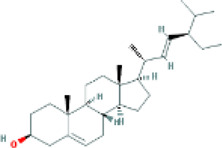	−10.1	
		2-Methoxy-9,10-dihydrophenanthrene-4,5-diol	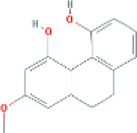	−8.4	
ESR1	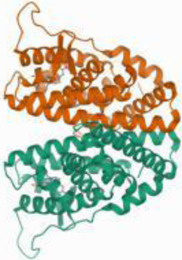	Beta-sitosterol	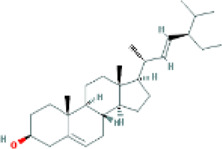	−7.3	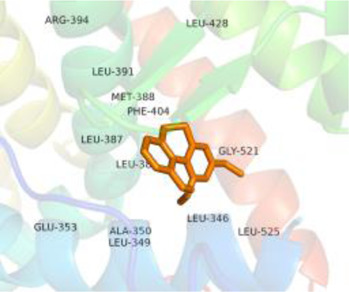
		Stigmasterol	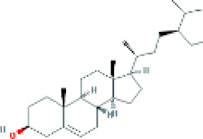	−6.8	
		2-Methoxy-9,10-dihydrophenanthrene-4,5-diol	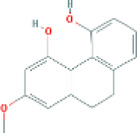	−8.4	
MTOR	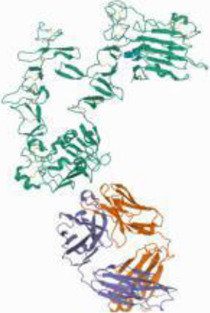	Beta-sitosterol	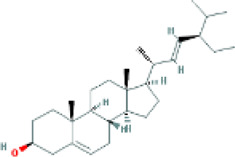	−5.0	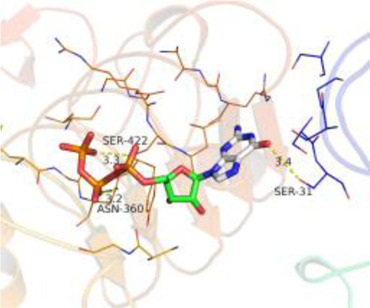
		Stigmasterol	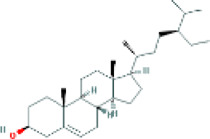	−5.0	
		2-Methoxy-9,10-dihydrophenanthrene-4,5-diol	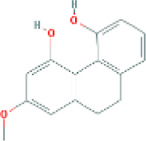	−5.0	
ERBB2	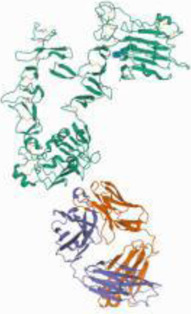	Beta-sitosterol	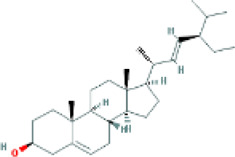	−4.7	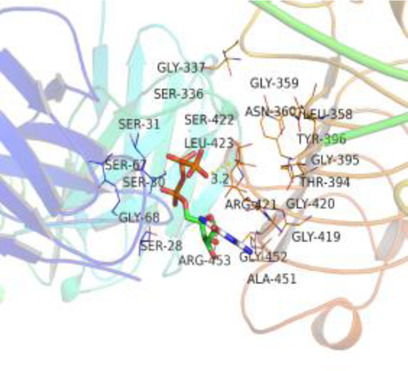
		Stigmasterol	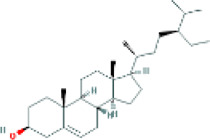	−5.1	
		2-Methoxy-9,10-dihydrophenanthrene-4,5-diol	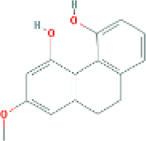	−5.0	
MCL1	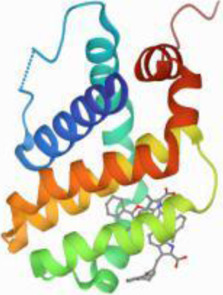	Beta-sitosterol	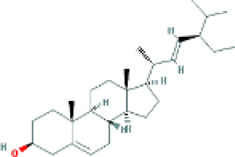	−6.9	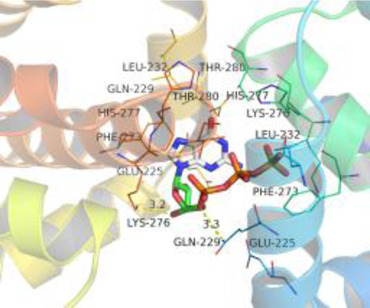
		Stigmasterol	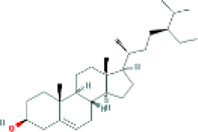	−6.8	
		2-Methoxy-9,10-dihydrophenanthrene-4,5-diol	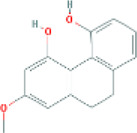	−6.7	
MMP2	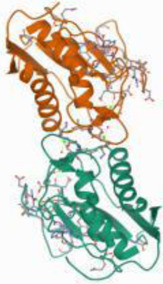	Beta-sitosterol	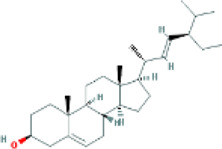	4.5	NA
		Stigmasterol	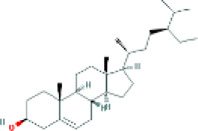	4.4	
		2-Methoxy-9,10-dihydrophenanthrene-4,5-diol	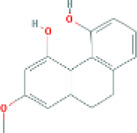	4.5	
MMP9	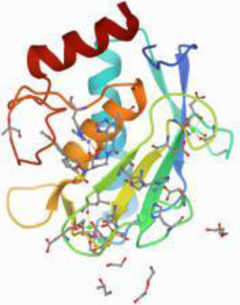	Beta-sitosterol	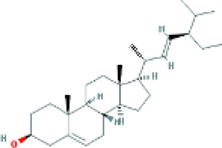	7.1	NA
		Stigmasterol	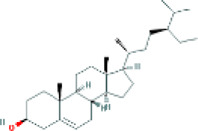	2.8	
		2-Methoxy-9,10-dihydrophenanthrene-4,5-diol	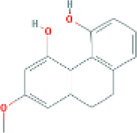	1.8	
KDR	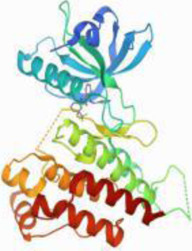	Beta-sitosterol	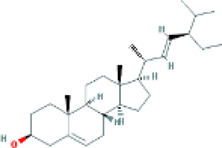	−2.8	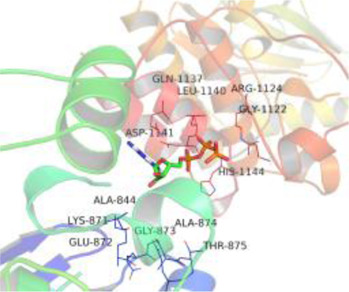
		Stigmasterol	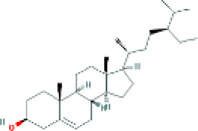	−2.7	
		2-Methoxy-9,10-dihydrophenanthrene-4,5-diol	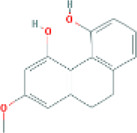	−3.3	
JAK2	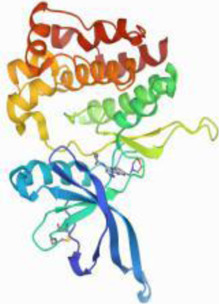	Beta-sitosterol	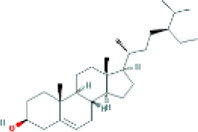	−1.8	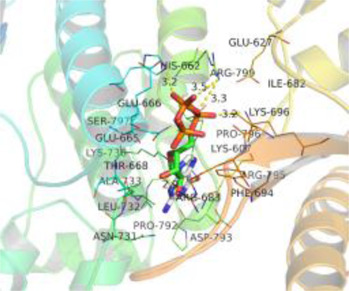
		Stigmasterol	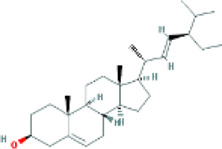	−1.9	
		2-Methoxy-9,10-dihydrophenanthrene-4,5-diol	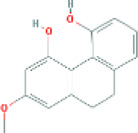	−1.7	

Note: The results of the docking data of MAPP2 and MAPP9 show that these two proteins cannot be binding sites; thus, NA means there is no optimal binding image.

### Survival Analysis

**FIGURE 8:**
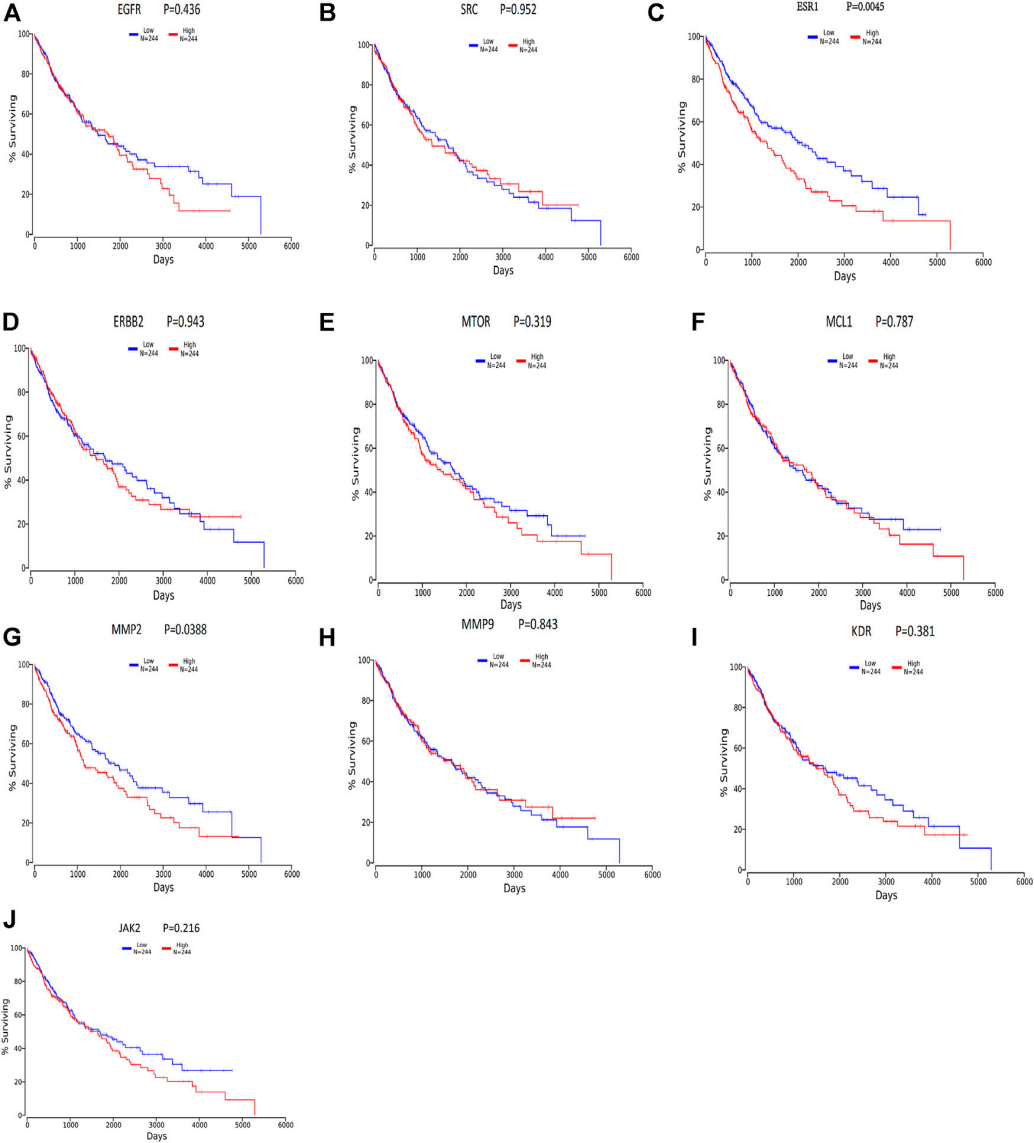
Survival analysis for hub genes by oncolnc. Red lines represent sample groups with high gene expression, while green lines represent sample groups with low gene expression.

We divided the lung squamous carcinoma cases into high and low expression groups based on the expression levels of ten core genes and investigated the correlation between the expression of each of the ten core genes and the prognosis of lung squamous carcinoma patients mainly using TCGA. As shown in Figure 8, highly expressed genes were associated with poor prognosis. Among the ten hub genes analyzed for survival, ESR1 (*p* = 0.00454) and MMP2 (*p* = 0.0388) were associated with overall survival in patients with lung squamous carcinoma (Figures 8C,G), and overall survival analysis of other hub genes with high and low expression did not show statistical significance (*p* < 0.05).

## Discussion

As of 2021, lung cancer will continue to be the leading cause of cancer death for both men and women, accounting for 22% of all cancer deaths. To further improve the survival rate of patients with lung cancer, this study identified the active ingredients and the possible detailed molecular mechanism of Shan Ci Gu in the treatment of NSCLC. This study supports the wider application and further mechanistic surveys of Shan Ci Gu for the treatment of NSCLC.

First, we aimed to derive the feasible active ingredients and targets according to the OB and DL values of the components of TCMSP. Consequently, three active components of Shan Ci Gu were obtained, and only 143 candidate targets of these active components were retrieved through Swiss Target Prediction. We constructed an herb-ingredient-target network, which reflected the relationship between numerous components and targets of the drug. In this study, three active components of Shan Ci Gu for the treatment of NSCLC were identified: β-sitosterol, stigmasterol, and 2-methoxy-9,10-dihydrophenanthrene-4,5-diol. Previous studies have mainly focused on anti-breast cancer, -colon cancer, and -prostate cancer effects of Shan Ci Gu. β-sitosterol, as a potential natural drug, can effectively prevent the occurrence and growth of many types of tumors. Zhou et al. (Zhou et al., 2016), in 2016, showed that β-sitosterol promotes the apoptosis of A549 cells in a dose-dependent manner in the range of 0–40 μM. The higher is the concentration of β-sitosterol, the higher is the apoptosis rate of the A549 cells. 2-Methoxy-9,10-dihydrophenanthrene-4,5-diol is a type of phenanthrene component, which has anti-tumor, anti-bacterial, anti-spasmodic, anti-inflammatory, anti-allergic, and anti-platelet aggregation biological activities. Xue et al. (Xue et al., 2006), in 2006, carried out cytotoxicity experiments that showed that blestriarene C and other phenanthrene components have strong inhibitory effects on the proliferation of HepG2 cells of liver cancer, and blestriarene A has certain inhibitory effects on the proliferation of A549 cells of lung cancer *in vitro*. Therefore, β-sitosterol, stigmasterol, and 2-methoxy-9,10-dihydrophenanthrene-4,5-diol are good anti-tumor agents, and related studies show that they are of importance in the proliferation and apoptosis of liver, colon, and breast cancer cells, while studies regarding NSCLC are scarce, and the mechanism of action of Shan Ci Gu remains unclear.

We retrieved 1,226 candidate NSCLC targets from the GeneCards database and 143 candidate targets of Shan Ci Gu through Swiss Target Prediction. We found 56 targets in common between the disease and Shan Ci Gu, which were considered potential targets for treating NSCLC. The degree of association between these genes is shown in Figures 3A,B. The top 10 core genes are shown in Figure 3C, including EGFR, ESR1, SRC, ERB2, MTOR, MCL1, MMP2, MMP9, KDR, and JAK2. These genes play a significant role in the proliferation, migration, and apoptosis of NSCLC cells. As EGFR and ERBB2 are human EGFRs, they have important impacts on the physiological processes of cell growth, proliferation, and differentiation in humans. Increased expression of EGFR is commonly observed in malignancies such as lung, breast, and pancreatic cancers, making this receptor a major target for the development of anti-tumor therapies (de Lavera et al., 2021). ESR1 and SRC are involved in endocrine regulation, and MMP2 and MMP9 regulate complex kinases, thus participating in cell proliferation and migration.

The target genes of Shan Ci Gu for the treatment of NSCLC were used to obtain an enrichment map of the GO and KEGG pathway analyses using R software (Figure 4). Figure 4 shows that the BP is mainly associated with the regulation of protein kinases such as MAP kinase and serine threonine kinase, and CC is mainly associated with various cell bodies such as the receptor complex, cell body, neuronal cell body, dendrite, and dendritic tree. The perinuclear region of cytoplasm MF is mainly associated with protein kinase activity. The main signaling pathways are the endocrine, EGFR-TKI resistance, ErbB, PI3K-Akt, and Rap1 signaling pathways. The diseases involved are prostate cancer and hepatitis B.

EGFR plays an important role in lung carcinogenesis, while its expression level is independent of EGFR positivity rate, lung cancer stage, lung cancer differentiation, the presence of lymph node metastasis, and so on. It should be used as an index of prognosis together with other cytokines. In human lung adenocarcinoma A549 cells, MMP secretes epidermal growth factor, activates the EGFR-ERK signaling pathway, and promotes the expression of claudin-2, thus promoting tumor colonization (ciardiello and Tortora, 2008). Among the EGFR-TKI resistance signaling pathways, KRAS is an important signaling pathway downstream of EGFR, and the mutated KRAS gene directly activates the MAPK signaling pathway without relying on the activation of upstream EGFR, leading to tumor proliferation and metastasis (Wang et al., 2020). Inhibition of EGFR expression can be used to treat NSCLC by modulating the immune microenvironment. This is because EGFR can upregulate immune checkpoints such as PD-L1 and IDO1, making NSCLC more resistant to drugs. In addition, ESR1 encodes an estrogen receptor involved in hormone binding, DNA binding, and transcription activation and participates in breast cancer, endometrial cancer, osteoporosis, and other pathological processes. ESR1 mRNA overexpression is associated with the prognosis of NSCLC (Teng et al., 2018). The existence of a ligand-independent ESR signaling pathway has been demonstrated, wherein ESR and its functional pathways are subject to multiple regulations such as those of the growth and differentiation of target cells, either through their own phosphorylation or through binding or interaction with different growth factors, co-regulators, oncogenes, or oncogenic proteins (Zhang et al., 2014). A study by Atmaca, in 2020, showed that assessment of ESR1 mRNA by qPCR is a feasible method to examine ESR1 expression in NSCLC, and ESR1 expression determines the prognosis of metastatic NSCLC. ESR1 is a predictive biomarker that is of therapeutic importance in breast cancer, and this study indicates that it can play a similar role in lung cancer (Atmaca et al., 2014). The Src tyrosine kinase inhibitor can be selectively used for the molecular targeting of NSCLC with high activation of Src proteins (Zheng et al., 2011). A study by Yao, in 2020, further demonstrated that hesperidin effectively inhibits the proliferation of NSCLC cells (A549 and H460) by inhibiting the SRC3-mediated ubiquitination of IGF-1R-PI3K-AKT signaling to induce apoptosis and exert an inhibitory effect on the tumor growth in NSCLC (Yao et al., 2020). Dong et al. found that quercetin inhibited the expression of Src, and subsequently, inhibited Fn14/NF-kappa B signaling, thereby suppressing the proliferation and metastasis of NSCLC. This suggests that Src expression promotes NSCLC progression and could be a target for the treatment of NSCLC (Dong et al., 2020). In the ErbB signaling pathway, the ErbB receptor family and its downstream pathways may regulate epithelial-mesenchymal transition, migration, and tumor invasion by regulating components of the extracellular matrix (ECM) (Kern et al., 1992). This mechanism occurs not only in NSCLC but also in other tumor growth processes such as those of breast, ovarian, and bladder cancer. EGFR is a member of the ErbB family and can bind to ECM components such as matrikines to promote tumor cell expansion (Appert-Collin et al., 2015). Yu et al., in 2020, found that TMPO-AS1 was upregulated in the cancerous tissues of NSCLC samples, which enhanced the expression of ERBB2, promoting the deterioration of NSCLC cells (Yu et al., 2020). The activation of mTOR causes an accelerated tumor cell cycle, shortened G1 phase duration, rapid cell proliferation, and increased secretion of on coproteins, and this promotes tumor development. mTOR acts in a synergistic manner to inhibit tumor growth in mouse prostate and lung cancer models, and phosphorylated or activated mTOR is found in 74% of the NSCLCs, making it an additional target for NSCLC therapy (Marinov et al., 2007). In A549 and primary human NSCLC cells, GDC-0349 inhibits NSCLC cell growth, proliferation, cell cycle progression, migration, and invasion through the Akt-Akt-mTOR pathway, while inducing significant apoptotic activation (Yang et al., 2020). Analysis of a receiver operating characteristic curve (area under curve = 0.6785) showed that the expression of MCL-1 is an important critical value for predicting prognosis in 30.0% of the NSCLC tumor cell types. Curcumin inhibits the expression of radiation-induced EMT and sE-cad by reducing the expression of MMP9, thereby inhibiting the migration and invasion of NSCLC (Deng et al., 2020). CCK8 and Transwell invasion assays have shown that A549 cells transfected with the miR-4448 inhibitor have higher proliferation and metastatic abilities. High expression of MMP2 and MMP9 in A549 cells transfected with the miR-4448 inhibitor has been confirmed by qRT-PCR and western blot (Xu et al., 2020). miR-142-3p overexpression inhibits the expression of NR2F6, MMP2, and MMP9 and improves caspase-3 expression, thereby inhibiting lung adenocarcinoma cell proliferation, migration, and invasion and enhancing apoptosis, demonstrating that miR-142-3p may be a new therapeutic target for lung adenocarcinoma treatment (Jin et al., 2019). In one study, compared to that in the normal lung cell line, miR-204 expression was found to be downregulated, while that of JAK2 was upregulated in four NSCLC cell lines (A549, H1299, H1650, and H358). These findings indicate that miR-204 functions as a tumor suppressor in NSCLC by acting on JAK2. Therefore, we can consider miR-204 as a biomarker for the diagnosis and treatment of NSCLC (Wang et al., 2016). Among the endocrine signaling pathways, the core targets EGFR, ERBB2, ESR1, MTOR, MMP2, MMP9, and SRC are in endocrine signaling pathways. Neuroendocrine dedifferentiation (NED) is widely found in tumors of prostate, gastrointestinal tract, and lung cancers, among others. Chen (Chen et al., 2014), have found that 0–20% of NSCLC is associated with NED and inhibition of the Akt signaling pathway. Clinical manifestations, natural course, pathological changes, and treatment response are all characteristic of NED and have become a new field of lung cancer research (Dudzińska et al., 2020). Ma et al. (Ma and Zhang, 2015), in 2015, found that with a positive rate of 72.3%, the expression of Rap1b was significantly higher in NSCLC tissues compared to that in paraneoplastic tissues. Further studies have confirmed that Rap1b is closely related to tumor differentiation, supporting the conclusion that Rap1b may have an oncogene function in the development of NSCLC. The results of survival analysis showed that the survival rate of the low-expression group of ESR1 and MMP2 was higher than that of the high-expression group, which further confirmed that reducing the expression of ESR1 and MMP2 could improve the quality of life of NSCLC patients.

## Conclusion

We speculate that Shan Ci Gu may play a role in inhibiting tumor cell proliferation by targeting several proteins such as EGFR, SRC, and ESR1 in NSCLC. The therapeutic effect of Shan Ci Gu involves a variety of BPs mainly involved in the inhibition of cell proliferation and endocrine effects, such as the endocrine, EGFR-TKI resistance, ErbB, and PI3K-Akt signaling pathways. In conclusion, Shan Ci Gu plays a role in the treatment of NSCLC through multiple targets and pathways. Therefore, the results of this study provide a basis for further research on the clinical application of Shan Ci Gu in NSCLC.

## Data Availability

The original contributions presented in the study are included in the article/Supplementary Material, further inquiries can be directed to the corresponding author.

## Abbreviations

TCMSP, Traditional Chinese Medicine Systems Pharmacology; OB, Oral bioavailability; DL, Drug likeness; NSCLC, Non-small cell lung cancer; PPI, Protein-protein interaction; STRING, Search Tool for the Retrieval of Interacting Genes/Proteins database; GO, Gene Ontology; BPs, Biological processes; MF, Molecular function; CC, Cellular component; KEGG, Kyoto Encyclopedia of Genes and Genomes; PDB, Protein Data Bank; SDF, Standard Delay Format; NED, Neuroendocrine dedifferentiation.
